# Predictive Models for Injury Risk Across Body Regions and Sport Types in Physically Active Students: Cross-Sectional Design

**DOI:** 10.3390/jcm14124307

**Published:** 2025-06-17

**Authors:** Jarosław Domaradzki, Edyta Kopacka

**Affiliations:** 1 Department of Biological Principles of Physical Activity, Wroclaw University of Health and Sport Sciences, 51-612 Wrocław, Poland; jaroslaw.domaradzki@awf.wroc.pl; 2 Independent Researcher, 51-612 Wrocław, Poland

**Keywords:** intrinsic risk factors, fat mass index, amateur athletes, injury prediction, sport disciplines, individual sports, team sports, body composition

## Abstract

**Background/Objectives**: Previous studies have typically investigated injury risk factors either by body region or sport type in isolation, limiting their practical applicability to real-world settings where multiple factors interact. However, injury risk is inherently multifactorial—shaped by a complex interplay of demographic, physiological, and training-related characteristics that differ by anatomical site and sport context. This study addresses that gap by simultaneously analyzing predictors across multiple body regions and sport-specific environments. This integrated approach is critical for developing more precise, evidence-based injury prevention strategies tailored to the specific demands and risk profiles of amateur athletes. This study aimed to identify key predictors of injury risk across various body regions and sport-specific contexts among amateur athletes. Specifically, we sought to (1) develop predictive models that include demographic and body composition variables, and (2) compare the relative predictive strength of these variables across models, highlighting differences in their influence by injury location and sport type. **Methods**: A total of 454 amateur athletes (219 males and 235 females) participated. Data on anthropometry, body composition, training load were collected. Injury history was obtained via self-administered questionnaires, with participants reporting injuries that had occurred during the 12 months prior to the time of data collection. Logistic regression models were used to identify significant predictors, and Nagelkerke’s R^2^ was calculated to assess model fit. **Results**: Overall, 49.78% of athletes experienced injuries, with a higher proportion in females (54.47%) than in males (44.75%). Age demonstrated divergent effects: it was protective against both upper and lower limb injuries in male individual-sport athletes (OR = 0.62 and OR = 0.69, respectively) and in female athletes across sport types (ORs = 0.75–0.64), but conversely increased the risk of upper limb injuries in male team-sport athletes (OR = 1.88). In female individual athletes, higher Skeletal Muscle Index (SMI) predicted upper limb injuries (OR = 1.18, *p* = 0.034). In female team athletes, higher Muscle-to-Fat Ratio (MFR) (OR = 2.46, *p* = 0.017) and BMI (OR = 1.67, *p* = 0.008) predicted upper limb injuries, while higher Fat Mass Index (FMI) predicted lower limb injuries (OR = 1.70, *p* = 0.009). Models showed moderate explanatory power (Nagelkerke’s R^2^ ranging from 0.03 to 0.33). **Conclusions**: These findings suggest that injury risk profiles are highly context-dependent. Preventive strategies should be tailored by sex and sport type, for example, younger athletes in team sports may benefit from age-sensitive load monitoring, while in female team athletes, targeted interventions addressing both fat and muscle balance could be essential. Age, body composition, and sport-specific demands should be considered in individualized injury prevention planning.

## 1. Introduction

Participation in sports and athletic activities entails a recognized risk of injury, driven by biomechanical loads and sport-specific movement patterns [1]. Understanding the mechanisms of injury is essential to inform prevention strategies [2]. While some injuries are inevitable, many can be mitigated through neuromuscular training, proper workload monitoring, and structured prevention programs [3,4,5]. Amateur athletes, particularly those without consistent medical or coaching support, are especially vulnerable to injury. The most commonly affected regions are the knee, ankle, and foot, with chronic overuse injuries prevailing over acute trauma [6,7,8]. Identified risk factors for sports-related injuries include sex, age, sport discipline, training load, and individual biomechanical characteristics such as asymmetries or faulty movement patterns [9,10]. These factors do not operate in isolation but interact in complex ways depending on the sport type and athlete profile. For instance, team sports typically exhibit a higher injury incidence than individual sports, largely due to the increased frequency of physical contact, dynamic play, and rapid directional changes [11,12]. In contrast, athletes involved in individual sports are more prone to overuse injuries, often resulting from repetitive strain and insufficient recovery time. Moreover, demographic factors like sex and age influence not only injury prevalence but also injury type—women have been shown to be more susceptible to certain non-contact and overuse injuries, while men tend to experience more acute trauma-related incidents. These patterns suggest that injury mechanisms and risk profiles are both sport-specific and gender-dependent, reinforcing the need for tailored prevention strategies that consider these contextual variables. Conversely, individual sports tend to be associated with overuse injuries stemming from repetitive strain. Fluctuations in training loads—particularly acute spikes—have also been strongly linked with increased injury rates [13,14,15].

Although injury surveillance in elite sports is well-developed, amateur athletes often lack access to medical monitoring, individualized coaching, or structured recovery protocols. As such, they represent a high-risk and under-studied population in terms of both prevention and early detection of musculoskeletal injuries. Unlike elite athletes, amateurs typically engage in sport alongside academic or professional commitments, often with inconsistent training routines and less biomechanical oversight. These factors contribute to unique injury profiles and necessitate dedicated study.

Despite extensive research into injury mechanisms and prevention strategies, gaps remain in our understanding of how risk factors interact across different athlete groups. Youth athletes, for instance, may be particularly susceptible to workload imbalances due to their ongoing development, with early sports specialization further increasing injury risk [16]. Most existing studies have focused on single variables (e.g., age or BMI alone), examined only one injury location or sport context at a time, or applied models with limited explanatory power—often failing to reflect the complex interplay between intrinsic and extrinsic risk factors [1,5,6]. Few studies have used multivariable predictive models to simultaneously assess injury risk across both body regions and sport types, especially in populations without elite-level training conditions. Moreover, many prior models report low explanatory strength (e.g., R^2^ < 0.10), leaving practical utility limited [12]. This study addresses those limitations by incorporating body composition indices (e.g., BMI, FMI, SMI, MFR) alongside training-related and demographic factors, and by stratifying models across sport types and injury sites to improve specificity and applicability [16]. Monitoring load and implementing age-appropriate conditioning have thus become essential preventive measures. Moreover, the distribution and severity of injuries can vary significantly between body regions and sport discipline types, suggesting the need for tailored prevention strategies. For example, for team game competitors, the critical body region is lower limbs, particularly in sports requiring high mobility and rapid directional changes [9], while practitioners of individual disciplines seem to suffer more often from upper limb injuries- Recent methodological advances such as overuse injury surveillance questionnaires [2] and validated functional screening tests provide enhanced tools for identifying at-risk athletes. Combined with body composition analysis, these tools can support the development of practical, targeted interventions—such as adjusting training loads in older team-sport athletes or monitoring fat–muscle balance in female populations—to reduce injury risk [12]. By identifying risk profiles early, practitioners can implement sex- and sport-specific strategies that improve training safety and long-term athlete development. To address these complexities, the present study aimed to identify the most relevant predictors of musculoskeletal injury across multiple body regions and sport-specific contexts in amateur athletes. Specifically, we aimed to (1) develop predictive models incorporating demographic and body composition variables and (2) assess and compare the strength of these predictors across sport types and injury locations. It was assumed that the occurrence of injuries, particularly in specific body regions and specific types, within the past year, could be meaningfully associated with current demographic, body composition, and training-related characteristics.

## 2. Materials and Methods

In this work, double frameset data was used to inform the number of participants sufficient to examine the predictive potential of the models regarding body parts and sport-specific types.

### 2.1. Ethics

All subjects gave their informed consent for inclusion before they participated in the study. The study was conducted in accordance with the Declaration of Helsinki, and the protocol was approved by the Ethics Committee of Wroclaw University of Health and Sport Sciences (consent number 13/2022, date of approval: 28 March 2022).

All participants were asked to provide informed consent via an online form prior to the study, and the purpose and characteristics of the research were explained to them.

### 2.2. Study Design

This study employs a cross-sectional design. This approach is the most common in the study of ex-post (retrospective) injury incidents. Examinations were conducted in 2022 and 2023 at Wroclaw University of Health and Sport Sciences among first-year students. Participants were asked to complete an online survey related to their injury history, followed by measurements of their anthropometric parameters and body composition. Two dataset frames have been merged. The predictor variables, including body composition and training characteristics, were assessed at the time of study participation, while the outcome variable—injury history—referred to the 12 months preceding data collection. This one-year retrospective interval was chosen to balance recall reliability and the likelihood of capturing a representative number of injury events. This time frame is consistent with previous injury surveillance studies in physically active populations and was selected based on both practical considerations (e.g., participant recall limits) and existing literature suggesting that a 12-month window provides sufficient data for modeling without excessive bias.

### 2.3. Sample Size

Before recruitment, a power calculation was conducted to determine the minimum sample size required for the logistic regression analysis. The rule of thumb was employed. Usually, a minimum of 10 participants per one variable in the model is suggested. However, we used a formula assuming the value of the margin of error (δ) [17]. The general formula for the sample size required to achieve a margin of error of δ in estimating a true probability of *p* at the 0.95 confidence level is:

$$n=(\frac{1.96}{\delta }{)}^{2}\times p\left(1-p\right).$$

Assuming a small margin of error of 0.0500, the maximum variance assumption *p* = 0.5 (worst-case probability), and 20% dropout, the total participant count was 462. These restrictive assumptions covered two sexes and up to ten independent variables.

### 2.4. Participants

**Figure 1:**
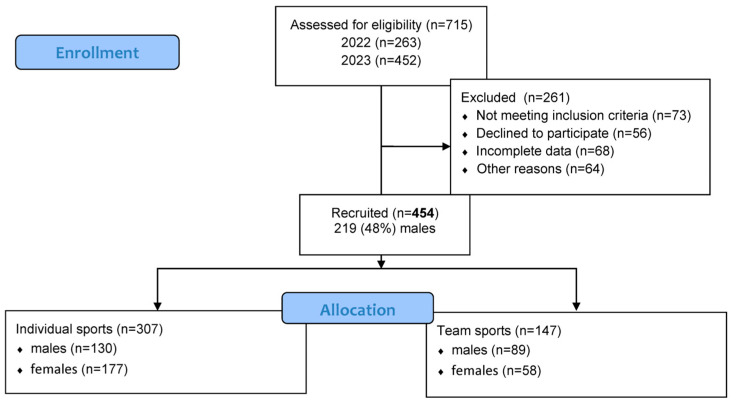
Flow diagram of the progress through all phases of data collection.

Participants included 454 healthy individuals, of which 219 (48%) were males. All participants were students at the Faculty of Physical Education and Sport and Physiotherapy in 2022 and 2023 at Wroclaw University of Health and Sport Sciences. The proportions of males and females represented the true population in these fields of study. The flowchart (Figure 1) presents the full sampling procedure. The structure of the studied group was: 130 males practicing individual sports and 89 males practicing team sports, 177 females practicing individual sports and 58 females practicing team sports.

Preliminary inclusion criteria required students to be active and attending classroom courses. The exclusion criteria included students participating in university-regulated sporting activities defined as institutionalized competitive training organized by the university’s sports clubs or the Academic Sports Association (AZS), as well as athletes enrolled in specialized sports programs or elite-level training classes or mastery-level programs, which involve enhanced training loads, specialized coaching, and competition preparation as part of their academic curriculum. These exclusions were applied to ensure the focus remained on non-elite amateur athletes whose training environment and injury exposure differed from that of professional or semi-professional athletes. A total of 454 students met the criteria and were accepted to participate in the examinations.

### 2.5. Anthropometric and Body Composition Measurements, Asymmetry Calculations

Anthropometric and body composition measurements were conducted in the Bi-okinetics Research Laboratory (part of the Central Research Laboratory) of Wroclaw University of Health and Sport Sciences. This facility holds Quality Management System Certificates PN-EN ISO 9001:2009 [18] (Certificate Reg. No.: PW-48606-10E) and PN-EN ISO 9001:2015 [19] (Certificate Reg. No.: PW-15105-22X).

Two body height measurements were taken with an accuracy of 0.1 cm using an anthropometer (GPM Anthropological Instruments, Dübendorf, Switzerland). Body weight and body fat weight were measured using a body composition analyzer with the InBody230 electronic tool (InBody Co. Ltd., Cerritos, CA, USA). Using body height, weight, and body fat mass, body mass index (BMI), fat mass index (FMI), skeletal muscle mass index (SMI) and muscle-to-body fat ratio (MFR) have been calculated using the following formulas:

$$\mathrm{BMI}=\frac{\mathrm{body}\;\mathrm{mass}\;\left[\mathrm{kg}\right]}{\mathrm{body}\;\mathrm{height}{\;[m}^{2}]}$$

$$\mathrm{FMI}=\frac{\mathrm{body}\;\mathrm{fat}\;\mathrm{mass}\;\left[\mathrm{kg}\right]}{\mathrm{body}\;\mathrm{height}{\;[m}^{2}]}$$

$$\mathrm{SMI}=\frac{\mathrm{body}\;\mathrm{skeletal}\;\mathrm{mass}\;\left[\mathrm{kg}\right]}{\mathrm{body}\;\mathrm{height}{\;[m}^{2}]}$$

$$\mathrm{MFR}=\frac{\mathrm{skeletal}\;\mathrm{muscle}\;\mathrm{mass}\;\left[\mathrm{kg}\right]}{\mathrm{body}\;\mathrm{fat}\;\mathrm{mass}\;\left[\mathrm{kg}\right]}$$

### 2.6. Recording of Musculoskeletal Injuries

Injury history was obtained via self-administered questionnaires, with participants reporting injuries that had occurred during the 12 months prior to the time of data collection. The Injury History Questionnaire (IHQ) was used to collect injury data. The reliability of the IHQ was previously assessed using Cronbach’s alpha analysis, with a calculated coefficient of 0.836 confirming high reliability [20,21]. The IHQ analyzes the number of injuries in the last 12 months, specifically concerning body parts. Injury information was self-reported. Students independently completed surveys using Google Forms. The completeness of the data was verified at the end, after compilation with physical measurements. As previously mentioned (and shown in Figure 1), there were no missing data on the injury questionnaire and among the 454 subjects selected for analysis.

### 2.7. Demographic and Training Load Characteristics

Independent variables used for prediction models were age and sex. Although, taking into account different values and effects of the body fat among males and females, all models were calculated for both sexes separately. Training load was calculated using information about frequency of the training during the week (days per week) multiplied by hours spent on training (hours per session). Independently, experience was also used in models as years of training.

### 2.8. Statistics

Continuous variables were expressed as mean, standard deviations and 95% confidence interval, while categorical variables were presented as numbers and frequencies.

Differences in continuous variables between groups were tested with two-way ANCOVA (sex and sport specificity as factors, and age as confounding variable). Detailed comparisons have been conducted with post-hoc tests using Bonferroni correction. Before analysis, an assumption testing procedure was conducted. Assumption testing for the two-way ANCOVA was conducted to ensure methodological rigor. Specifically, the assumptions of normality of residuals (via the Shapiro–Wilk test), homogeneity of variance (via Levene’s test), and homogeneity of regression slopes (via interaction testing between the covariate and independent variables) were verified. No violations of these assumptions were detected. In cases where mild deviations occurred, sensitivity analyses were performed, and the results remained consistent, supporting the robustness of the findings.

Differences in frequencies between factors’ categories (sex, sport specificity, body parts) have been tested with the chi-squared test, with Yates correction and φ as an effect size and OR with 95% CI.

To identify significant predictors of injury occurrence, stepwise backward logistic regression models were constructed, with injury status (1 = injury occurred, 0 = not occurred) as the dependent variable and age, BMI, FMI, SMI, MFR, training load, and training experience as independent variables. Prior to regression analysis, all predictors were tested for multicollinearity using Variance Inflation Factor (VIF), with a VIF threshold of <5 indicating acceptable levels of collinearity. To assess goodness-of-fit, as well as differences between models, various statistics were calculated: Wald’s statistics, likelihood ratio test, and AUC. Differences between the AUC of the models were tested with the DeLong test, while the differences in power between the same variables across different models were tested with the z-statistic for β differences and OR significance of the different approaches. Specifically, the following formulas were used:

$$z=\frac{\beta_{1}-\beta_{2}}{\sqrt{{SE}_{1}^{2}-{SE}_{2}^{2}}},\;\mathrm{and}\;z=\frac{log({\mathrm{OR}}_{1})-log({\mathrm{OR}}_{2})}{\sqrt{SE(log{\mathrm{OR}}_{1}{)}^{2}-SE(log{\mathrm{OR}}_{2}{)}^{2}}}$$

The significance level for all statistical tests and procedures was set at an α-value of 0.05. Calculations were conducted using Statistica 13.5 (StatSoft Poland 2023, Cracow, Poland).

## 3. Results

### 3.1. Baseline Characteristics and Injury Frequencies

Descriptive statistics of age, anthropometric measurements, body composition indices and training characteristics (weekly load and experience) per sex and sport-specific type are presented in Table 1. ANCOVA results confirmed the significant main effect of the sex (Wilk’s Lambda = 0.230, F = 186.00, *p* < 0.001, η^2^_part_ = 0.77), but not sport specificity (Wilk’s Lambda = 0.984, F = 1.00, *p* < 0.519, η^2^_part_ = 0.02). However, significant interaction sex × sport specificity indicated the effect of the sex disappeared in the case of some variables (Wilk’s Lambda = 0.9554, F = 3.00, *p* < 0.009, η^2^_part_ = 0.04). Post hoc tests with Bonferroni correction revealed all between-sex differences in anthropometric measurements and all body composition indices (all *p* < 0.001), but not in weekly training load (all *p* > 0.05). In the case of sports experience expressed by years of sport practice, there were no significant differences between males practicing individual sports and both females’ groups: individual and team sports (*p* = 0.956 and *p* = 0.066, respectively), while males practicing team sports differed from both females groups (both *p* < 0.001).

Out of 454 participants, 226 (49.78%) suffered from musculoskeletal injuries. Considering sex, out of 219 males, 98 (44.75%) suffered from musculoskeletal injuries, while out of 235 females, 128 (54.47%) had experienced at least one injury during the 1 year before the questionnaire (Table 2). For the rest of the participants, both sexes (males: n = 121, 55.25%; females: n = 107, 45.53%) did not suffer any musculoskeletal injury. However, the chi-squared test results showed no significant differences neither in sex proportions (χ^2^Yates = 1.33, *p* = 0.248, φ = −0.08, OR = 0.73 [0.42–1.25%CI]), nor in sport specificity proportions (χ^2^Yates = 0.03, *p* = 0.857, φ = 0.12, OR = 1.06 [0.58–1.9%CI]).

Detailed frequencies of the injuries regarding body parts are presented in Table 3. Out of 219 males, 130 individuals (59.36%) practiced individual sports, while 89 (40.64)—team sports (Table 3). Out of 235 females, 177 (75.32%) practiced individual sports, while 58 (24.68)—team sports. Out of the males who practiced individual sports, 17 (13.08%) suffered from musculoskeletal injuries of the head, neck or trunk, while among the males who practiced team sports—9 (10.11%) individuals. These proportions have not been significant (χ^2^Yates = 0.21, *p* = 0.650, OR = 0.75 [0.32–1.76%CI]). Out of the females who practiced individual sports, 23 (12.99%) suffered from musculoskeletal injuries of the head, neck or trunk, while among the females who practiced team sports—6 (10.34%) individuals. These proportions are not significant (χ^2^Yates = 0.28, *p* = 0.594, OR = 0.77 [0.30–2.00%CI]).

Out of the males who practiced individual sports, 33 (25.38%) suffered from musculoskeletal injuries of the upper limb, while among the males who practiced team sports—18 (20.22%) individuals (Table 3). These proportions have not been significant (χ^2^Yates = 0.79, *p* = 0.375, OR = 0.75 [0.39–1.43%CI]). Out of the females who practiced individual sports, 32 (18.08%) suffered from musculoskeletal injuries of the upper limb, while among the females who practiced team sports—12 (20.69%) individuals. These proportions are not significant (χ^2^Yates = 0.20, *p* = 0.658, OR = 1.18 [0.56–2.48%CI]).

Out of males who practiced individual sports, 64 (49.23%) suffered from musculoskeletal injuries of the lower limb, while among the males who practiced team sports—40 (44.94%) individuals (Table 3). These proportions are not significant (χ^2^Yates = 0.39, *p* = 0.532, OR = 0.84 [0.49–1.45%CI]). Out of the females who practiced individual sports, 70 (39.55%) suffered from musculoskeletal injuries of the lower limb, while among the females who practiced team sports—24 (41.38%) individuals. These proportions have not been significant (χ^2^Yates = 0.06, *p* = 0.805, OR = 1.07 [0.59–1.97%CI]).

Although none of the comparisons reached statistical significance, reporting these results contributes to the overall understanding of injury distribution patterns across sport types. The consistently non-significant findings suggest that, in this sample of physically active students, sport type alone may not be a decisive factor in determining the occurrence of upper or lower limb musculoskeletal injuries. This reinforces the need for multivariable modeling to uncover more nuanced predictors that interact with other factors, such as age, body composition, and training experience.

### 3.2. Predictive Modeling

Studying the male groups, among the analyzed models, only age was identified as a statistically significant predictor of injury risk. In the male individual group, age was a protective factor for both upper limb and lower limb injuries. Specifically, each additional year of age was associated with a 38% reduction in the odds of upper limb injuries (OR = 0.62, 95% CI: 0.47–0.84, *p* = 0.002) and a 31% reduction in the odds of lower limb injuries (OR = 0.69, 95% CI: 0.56–0.86, *p* = 0.001) (Table 4). The models for upper and lower limb injuries in this group demonstrated moderate explanatory power, with Nagelkerke’s R² values of 0.14 and 0.13, respectively.

In contrast, among male team athletes, age was a significant risk factor for upper limb injuries, with each additional year of age increasing the odds by 88% (OR = 1.88, 95% CI: 1.14–3.10, *p* = 0.013) (Table 4). This finding suggests a differential impact of aging on injury risk depending on the type of athletic participation. The model for upper limb injuries in this group exhibited the strongest fit, with Nagelkerke’s R^2^ = 0.33, indicating that a larger proportion of the variance was explained compared to other models.

No other variables, including FMI, MFR, SMI, or BMI, reached statistical significance in any of the models. Additionally, the AUC values for significant predictors ranged from 0.66 to 0.78, indicating moderate discriminative ability in predicting injury risk (Table 4). The contrasting effects of age on injury risk between individual and team athletes suggest that different mechanisms may underlie injury susceptibility in these groups, potentially influenced by training loads, competition dynamics, or recovery patterns.

Among female athletes, statistically significant predictors of injury risk varied across body regions and participation types. In the female individual group, Skeletal Muscle Index (SMI) was a significant risk factor for upper limb injuries (*β* = 0.17, OR = 1.18, *p* = 0.034), indicating that higher SMI was associated with an increased likelihood of injury (Table 4). The model fit was relatively low (Nagelkerke’s R^2^ = 0.08), suggesting that other unmeasured factors may play a role. Additionally, age was a protective factor for lower limb injuries (*β* = −0.29, OR = 0.75, *p* = 0.011), with increasing age reducing the likelihood of injury. However, the model explained a limited proportion of variance (Nagelkerke’s R^2^ = 0.07), and AUC values suggested weak discriminatory ability.

In the female team group, Muscle-to-Fat Ratio (MFR) and BMI were significant risk factors for upper limb injuries. A higher MFR was associated with a 2.46-fold increase in the odds of upper limb injuries (OR = 2.46, *p* = 0.017), while higher BMI was also a significant risk factor (OR = 1.67, *p* = 0.008) (Table 4). The model showed stronger predictive power than the individual athlete models, with Nagelkerke’s R^2^ = 0.27, suggesting a better explanation of injury risk factors. For lower limb injuries, age was again a protective factor (OR = 0.64, *p* = 0.043), while Fat Mass Index (FMI) was a strong risk factor (OR = 1.70, *p* = 0.009). This model exhibited a relatively high explanatory power (Nagelkerke’s R^2^ = 0.26) and moderate predictive accuracy based on AUC values.

The contrasting role of age between individual and team athletes, as well as the influence of body composition variables on upper and lower limb injuries, suggests distinct injury mechanisms between these groups. Higher muscle mass and BMI were associated with greater injury risk in team athletes, potentially due to increased physical demands or contact exposure, whereas age was a protective factor across all groups for lower limb injuries. These findings highlight the importance of considering sport-specific and physiological differences in injury risk assessments.

### 3.3. Evaluating the Relative Impact of Predictors

In male athletes, comparisons between models revealed significant differences in the effect of age. In individual athletes, age was a protective factor for upper and lower limb injuries, with each additional year reducing the odds by 38% (OR = 0.62, *p* = 0.002) and 31% (OR = 0.69, *p* = 0.001), respectively. In team athletes, however, age was a risk factor for upper limb injuries, increasing the odds by 88% (OR = 1.88, *p* = 0.013) (Table 5).

In female athletes, comparisons between models revealed significant differences in predictor effects. Age and body composition variables played distinct roles in injury risk. In individual athletes, age was a protective factor for lower limb injuries, reducing the odds by 25% (OR = 0.75, *p* = 0.011). In team athletes, age remained protective for lower limb injuries (OR = 0.64, *p* = 0.043), but FMI was a significant risk factor, increasing the odds by 70% (OR = 1.70, *p* = 0.009). Additionally, for upper limb injuries in team athletes, MFR and BMI were significant risk factors, increasing the odds by 146% (OR = 2.46, *p* = 0.017) and 67% (OR = 1.67, *p* = 0.008), respectively.

## 4. Discussion

The aim of this study was to identify injury predictors based on gender and sport specificity (individual vs. team sports), considering age, anthropometric characteristics, body composition, and training characteristics. Our findings reveal notable sex- and discipline-specific differences in sports injury occurrence and predictors. Women reported a higher overall injury rate than men in the year preceding the study. Among men, injury prevalence varied by sport type, with more injuries occurring in team sports compared to individual sports. In contrast, injury rates among women were unrelated to sport type, although those in team sports sustained more injuries than those in individual disciplines. Across both sexes, the lower limbs were most injured, followed by upper limbs, and then head-neck-trunk areas. In men, injuries to all body regions were more frequent in individual sports, whereas in women, the reverse was observed. Age emerged as a key predictor: among male individual athletes, it was protective against upper and lower limb injuries, while in male team athletes, older age increased the risk of upper limb injuries (OR = 1.88, *p* = 0.013). For women, age was protective for lower limb injuries across both sport types, whereas higher fat mass index (FMI) increased the risk in team sports. Additionally, muscle-to-fat ratio (MFR) and BMI were significant risk factors for upper limb injuries in female team athletes. The highest predictive accuracy (AUC = 0.78) was observed in the model for upper limb injuries in male team athletes, and the best-fitting models for head-neck-trunk injuries in both sexes included BMI and years of training experience. These results highlight the complex interplay between sex, age, body composition, and sport type in determining injury risk.

Women reported a higher overall injury rate than men (54.47% vs. 44.75%) in the year preceding the study. Among men, injuries were more prevalent in team sports compared to individual sports (49.44% vs. 41.54%), while among women, injury rates were not significantly related to sport type, although those in team sports had a slightly higher prevalence. Across both sexes, the lower limbs were most frequently injured, followed by the upper limbs and then the head-neck-trunk region. These findings support the well-established pattern of lower limb vulnerability but offer a new perspective by contextualizing it through body region and sport-specific lenses. In contrast to a previous meta-analysis [22], which reported a higher overall injury incidence in male team-sport athletes (IRR = 0.86) and a greater risk of ACL injuries in females, our study found that women experienced significantly more injuries overall than men. Among men, injury prevalence was significantly higher in team sports compared to individual sports (49.44% vs. 41.54%, χ^2^ = 4.12, *p* = 0.042, OR = 1.39 [95% CI: 1.01–1.91]). However, among women, the difference between sport types was not statistically significant (χ^2^ = 0.15, *p* = 0.699). Likewise, a referenced study [23] demonstrated sex differences in injury patterns—men showed a higher incidence of acute injuries (49.8 vs. 38.6 per 10,000 AEs), while women had a higher rate of overuse injuries (24.6 vs. 13.2 per 10,000 AEs). Both studies suggest that although injury mechanisms differ between sexes, women may still be more susceptible to certain types of injuries, potentially explaining the higher overall injury rate observed in our female athletes.

Age played a different role depending on gender and sport type. In men, age emerged as a consistent predictor. It was protective against upper and lower limb injuries in individual sports (OR = 0.62–0.69), possibly due to accumulated experience, improved self-regulation, and more effective load management [24]. However, in male team athletes, age increased the risk of upper limb injuries (OR = 1.88), potentially reflecting cumulative exposure to high-impact movements and inadequate recovery protocols. These contrasting effects suggest that age alone is not a universal indicator of injury risk, but its impact is modulated by sport-specific physical demands. Correspondingly to our findings, which identified age as a significant risk factor for upper limb injuries in male team sport athletes, previous studies also suggest an age-related increase in injury risk. Reference [25] observed that older adolescent elite athletes reported a higher proportion of injuries compared to younger athletes. The divergent effects of age in individual versus team sports may be attributed to different physical demands and pacing strategies. In individual sports, accumulated experience likely supports self-regulation and injury prevention, while in team sports, prolonged exposure to high-contact, high-velocity play may exacerbate wear and tear, especially without adequate recovery protocols [26]. These findings are consistent with the observed trend in our study, where age was associated with a higher risk of upper limb injuries among male team sport athletes. Among women, age consistently served as a protective factor against lower limb injuries, both in individual (OR = 0.75, *p* = 0.011) and team sports (OR = 0.64, *p* = 0.043). Reference [27] suggests that this is due to improved postural control and landing biomechanics with increasing age.

In women, body composition indicators had a stronger influence than age. In team sports, high FMI predicted lower limb injuries (OR = 1.70), while MFR and BMI predicted upper limb injuries (OR = 2.46 and OR = 1.67, respectively). These results may be partly explained by biomechanical factors such as altered movement patterns due to increased fat mass or disproportionate strength development. Sociocultural aspects, such as limited access to targeted conditioning or different perceptions of pain and injury, may also contribute. This is consistent with findings by [28], which investigated the incidence and risk factors of medial tibial stress syndrome (MTSS) in physical education students. The study identified above-average Body Mass Index (BMI) as a significant risk factor for MTSS, with an odds ratio of 2.29 (95% CI 1.02 to 5.16). This finding underscores the role of body composition, particularly higher BMI, in increasing the risk of lower limb injuries among female athletes.

For upper limb injuries in female team athletes, MFR and BMI were significant risk factors (OR = 2.46 and OR = 1.67, respectively). This result aligns with this study [29]. Both studies consistently point out that, in female athletes, MFR and BMI are significant predictors of injury risk. While the cited study indicates these indices as general predictors for musculoskeletal injuries in women, our findings specifically highlight their role in predicting upper limb injuries among female team-sport athletes.

In men, body composition predictors (FMI and BMI) were significant only in models related to head-neck-trunk injuries, which was also observed by [30]. These results may reflect physiological and sociocultural differences; for instance, higher fat mass in female athletes may alter joint loading or biomechanics during multidirectional movements typical of team sports. Additionally, body composition may affect self-perception, movement confidence, or fatigue resistance, indirectly influencing injury likelihood. Research suggests that physiological and sociocultural factors contribute to sex differences in athletic performance and injury risk. Body composition plays a role in predicting sports injuries, with components like body mass index, weight, and bone density identified as risk factors [31]. Female athletes may have higher fat mass, potentially altering joint loading and biomechanics during multidirectional movements [32]. However, the relationship between physical fitness attributes and sports injury in female team ball sport players remains unclear, with mixed or insufficient evidence for most fitness components [33]. These findings highlight the complex interplay between physiological, biomechanical, and sociocultural factors in athletic performance and injury risk, emphasizing the need for further research in this area.

The comparative analysis revealed that, among men, age acts as a protective factor in individual sports but increases the risk of injuries in team sports. In women, body composition components (FMI, BMI, MFR) and age were key predictors, differentiating injury risks depending on injury location and sport specificity. Younger age and greater experience are associated with increased injury rates, particularly in team sports [34]. However, team sport athletes tend to have longer careers, retiring later and combining sports with work more effectively than individual sport athletes. Individual sport athletes train more intensively and face greater challenges during retirement transition [26]. Interestingly, while team sports show higher overall injury rates, the prevalence of overuse injuries appears higher in individual sports, with a pooled period-prevalence of 42% compared to 33% in team sports [6].

Our findings contrast with a meta-analysis [22] reporting a higher overall injury incidence in male team-sport athletes and greater ACL injury risk in females. In contrast, our data showed higher overall injury rates in women and sport-type-related differences only in men. Equally, [23] reported that women experience more overuse injuries, supporting our observed higher injury burden in female participants. Furthermore, [28] identified above-average BMI as a significant risk factor for MTSS, reinforcing the link between fat mass and lower limb injury in women. Our findings also mirror results from [29,30], which associated MFR and BMI with musculoskeletal injury risk in female athletes.

Our study also identified lower limb injuries, particularly involving the ankle, as the most common among athletes, followed by upper limb and head-neck-trunk injuries. However, unlike the reviewed studies where ankle injuries dominated, our results indicated that, overall, lower limb injuries (without specifying the ankle) were the most frequent across both sexes. Moreover, while the review highlighted a higher injury rate during games than practices, our study did not distinguish injury rates based on activity type.

While the current models accounted for demographic, body composition, and training-related variables, other important factors may have influenced injury risk but were not assessed in this study. Specifically, training surfaces (e.g., artificial turf vs. hardwood), biomechanical asymmetries (such as limb dominance or postural deviations), and recovery protocols (e.g., frequency of rest days, sleep hygiene, or use of regeneration techniques) are all known to affect injury susceptibility. Their absence from the present models may have limited the overall explanatory power and should be considered in future research. Incorporating such variables may enhance the predictive accuracy of injury models and offer more comprehensive insights for injury prevention strategies.

This study has several limitations. Injury data were collected cross-sectionally, retrospectively through self-reported questionnaires, which may have introduced recall bias and inaccuracies. Key factors such as previous injury history, detailed biomechanical profiles, and recovery strategies were not included, which could have limited the scope of the predictive models. One notable limitation of this study is the uneven sample sizes across subgroups, particularly the relatively small number of female participants in team sports (n = 58). This imbalance may reduce the statistical power and stability of estimates in subgroup comparisons, increasing the risk of both Type II errors (failing to detect true effects) and inflated confidence intervals. Consequently, non-significant findings in smaller groups should be interpreted with caution, as they may reflect limited power rather than a true absence of effect. The models demonstrated moderate predictive power (Nagelkerke’s R^2^ ranging from 0.03 to 0.33), suggesting that additional variables likely influence injury occurrence. Furthermore, the study did not distinguish between injuries sustained during training versus competition, which could have provided more nuanced insights. Finally, as the study sample consisted of amateur athletes, mainly students practicing different sports disciplines, caution should be exercised when generalizing the findings to elite athletic populations. Another limitation is the high exclusion rate of potential respondents. Additionally, the lack of data on biomechanical, environmental, and recovery-related factors may have introduced unmeasured confounding. Future research should adopt prospective designs and integrate objective biomechanical and training load data to improve model accuracy.

## 5. Conclusions

This study demonstrated that musculoskeletal injury risk among amateur athletes is shaped by a complex interplay between sex, sport type, body region, and individual characteristics such as age and body composition. The findings challenge simplified or one-size-fits-all approaches to injury prevention, highlighting that risk factors are not universal but instead group-specific and context-dependent. From a theoretical standpoint, the divergent role of age in men—protective in individual sports yet risk-enhancing in team settings—suggests that cumulative exposure, rather than chronological age alone, may better explain injury susceptibility. This points to the need for more nuanced models of age-related injury risk that account for sport-specific intensity, recovery profiles, and motor adaptation processes. Among female athletes, the prominence of body composition indicators (FMI, MFR, BMI)—especially in team sports—supports emerging evidence that fat and muscle distribution play critical roles in neuromuscular control and injury mechanics. These findings underscore the clinical value of incorporating body composition screening into preseason evaluations and injury prevention planning, particularly for women in multidirectional sports.

These findings highlight the need for injury prevention strategies that are not only sport-specific but also tailored to the unique physiological and biomechanical characteristics of male and female athletes. In male team athletes, priority should be given to managing cumulative training loads, ensuring recovery quality, and integrating age-sensitive periodization models. In female team athletes, optimizing body composition through strength, conditioning, and nutrition interventions could reduce injury risk, especially for upper limbs. For both sexes, identifying sport- and region-specific vulnerabilities enables the design of personalized screening and monitoring systems that go beyond general fitness assessments. Training programs should therefore incorporate sex-specific screening, strength and conditioning tailored to body composition, and education on injury mechanisms to support safer athletic participation. Finally, this study contributes to a more differentiated understanding of injury epidemiology in amateur populations—an often overlooked group lacking institutional medical support. These insights can inform coaches, sports educators, and clinicians working with non-elite athletes, and also lay the groundwork for future prospective studies, which should incorporate biomechanical, environmental, and recovery-related variables for improved predictive precision.

## Figures and Tables

**Table 1:** Overview of participants’ age, anthropometrical measurements, body composition indices, and training characteristics.

Variable	Males				Females			
	Mean	−95%CI	95%CI	sd	Mean	−95%CI	95%CI	sd
				individual				
Age [years]	21.8	21.5	22.1	1.9	21.2	21.0	21.4	1.5
BH [cm]	182.2	181.0	183.4	7.0	167.9	167.1	168.8	5.8
BW [kg]	80.3	78.3	82.2	11.1	60.3	59.1	61.5	8.3
BMI [kg/m^2^]	24.1	23.7	24.6	2.7	21.4	21.0	21.8	2.6
FMI [kg/m^2^]	4.0	3.7	4.2	1.4	5.1	4.8	5.3	1.7
SMI [kg/m^2^]	16.7	16.1	17.3	3.6	10.3	9.9	10.8	3.0
MFR [score]	4.9	4.5	5.2	2.0	2.7	2.5	2.8	1.0
Load [h/week]	6.6	5.9	7.3	4.1	5.6	5.0	6.2	3.8
Experience [y]	3.4	3.2	3.6	1.3	3.2	3.0	3.4	1.5
				team				
Age [years]	21.8	21.4	22.2	1.9	21.2	20.8	21.6	1.5
BH [cm]	181.9	180.5	183.4	7.1	169.3	167.5	171.0	6.6
BW [kg]	78.1	76.5	79.7	7.6	62.7	60.1	65.3	10.0
BMI [kg/m^2^]	23.6	23.2	24.0	2.0	21.8	21.1	22.6	2.8
FMI [kg/m^2^]	3.8	3.5	4.0	1.2	5.3	4.8	5.8	1.9
SMI [kg/m^2^]	16.2	15.6	16.8	3.0	11.5	10.8	12.2	2.6
MFR [score]	4.9	4.5	5.3	1.9	2.9	2.6	3.2	1.2
Load [h/week]	5.8	5.0	6.5	3.5	5.8	4.8	6.8	3.9
Experience [y]	3.9	3.7	4.2	1.2	2.9	2.5	3.2	1.5

Footnote: BH—body height, BW—body weight, BMI—body mass index, FMI—fat mass index, SMI—skeletal muscle mass index, MFR—muscle mass to fat ratio, load—training load, experience—years of training.

**Table 2:** Numbers and percentages of the injured participants by sex and sport specificity.

Sex			Injury N (%)	
	Sport	1	0	All
males	individual	54 (41.54%)	76 (58.46%)	130 (42.35%)
	team	44 (49.44%)	45 (50.56%)	89 (60.54%)
	whole	98 (44.75%)	121 (55.25%)	219 (48.24%)
females	individual	97 (54.80%)	80 (45.20%)	177 (57.65%)
	team	31 (53.45%)	27 (46.55%)	58 (39.46%)
	whole	128 (54.47%)	107 (45.53%)	235 (51.76)
all		226 (49.78)	228 (50.22)	454

**Table 3:** Numbers and percentages of the injured participants by sex, sport specificity and body parts.

Sex	Sport	All	Head-Neck-Trunk 0	Head-Neck-Trunk 1	Upper Limb 0	Upper Limb 1	Lower Limb 0	Lower Limb 1
Males	individual	130 (42.35%)	113 (86.92%)	17 (13.08%)	97 (74.62%)	33 (25.38%)	66 (50.77%)	64 (49.23%)
	team	89 (60.54%)	80 (89.89%)	9 (10.11%)	71 (79.78%)	18 (20.22%)	49 (55.06%)	40 (44.94%)
	whole	219	193 (88.13%)	26 (11.87%)	168 (76.71%)	51 (23.29%)	115 (52.51%)	104 (47.49%)
females	individual	177 (57.65%)	154 (87.01%)	23 (12.99%)	145 (81.92%)	32 (18.08%)	107 (60.45%)	70 (39.55%)
	team	58 (39.46%)	52 (89.66%)	6 (10.34%)	46 (79.31%)	12 (20.69%)	34 (58.62%)	24 (41.38%)
	whole	235	206 (87.66%)	29 (12.34%)	191 (81.28%)	44 (18.72%)	141 (60.00%)	94 (40.00%)
all		454	399 (87.89%)	55 (12.11%)	359 (79.07%)	95 (20.93%)	256 (56.39%)	198 (43.61%)

**Table 4:** Models containing variables most predictive of risk of injury in body parts—results of the backward stepwise logistic regression. Significant coefficients or those very close to significance are presented in bold.

Body Part	Variable	Beta	SE	Wald	*p*	OR	–95%CI	+95%CI	LRT
				Males	individual				
H-n-tr	MFR	0.28	0.19	2.19	0.139	1.33	0.91	1.93	−49.93
	FMI	0.30	0.28	1.20	0.274	1.35	0.79	2.32	−49.36
Model Fit Statistics: AIC = 104.73, BIC = 113.32, Nagelkerke’s R^2^ = 0.03 || AUC MFR = 0.55, AUC FMI = 0.48, Δ AUC = −0.07, *p* = 0.661									
Upper limb	Age	−0.47	0.15	9.92	**0.002**	0.62	0.47	0.84	−67.93
	FMI	0.18	0.15	1.47	0.226	1.20	0.89	1.62	−67.19
Model Fit Statistics: AIC = 140.39, BIC = 148.99, Nagelkerke’s R^2^ = 0.14 || AUC Age = 0.68, AUC FMI = 0.51, Δ AUC = −0.17, *p* = **0.064**									
Lower limb	Age	−0.37	0.11	11.17	**0.001**	0.69	0.56	0.86	−84.00
	FMI	0.12	0.13	0.77	0.379	1.13	0.86	1.47	−83.61
Model Fit Statistics: AIC = 173.22, BIC = 181.82, R^2^ Nagelkerke = 0.13 || AUC Age = 0.66, AUC FMI = 0.49, Δ AUC = −0.17, *p* = **0.040**									
				**Males**	**team**				
H-n-tr	FMI	−0.73	0.45	2.57	0.109	0.48	0.20	1.18	−28.86
	BMI	0.42	0.26	2.53	0.112	1.52	0.91	2.54	−27.55
Model Fit Statistics: AIC = 61.10, BIC = 68.57, Nagelkerke’s R^2^ = 0.07 || AUC FMI = 0.59, AUC BMI = 0.43, Δ AUC = −0.16, *p* = **0.055**									
Upper limb	Age	0.63	0.26	6.11	**0.013**	1.88	1.14	3.10	−36.17
	SMI	−0.28	0.16	3.14	0.077	0.75	0.55	1.03	−34.32
Model Fit Statistics: AIC = 74.65, BIC = 82.11, Nagelkerke’s R^2^ = 0.33 || AUC Age = 0.78, AUC SMI = 0.74, Δ AUC = −0.04, *p* = 0.438									
Lower limb	FMI	−0.30	0.19	2.60	0.107	0.74	0.51	1.07	−59.93
	SMI	0.09	0.08	1.42	0.233	1.09	0.94	1.27	−59.20
Model Fit Statistics: AIC = 124.41, BIC = 131.88, Nagelkerke’s R^2^ = 0.06 || AUC FMI = 0.38, AUC SMI = 0.43, Δ AUC = 0.05, *p* = 0.589									
				**Females**	**individual**				
H-n-tr	Load	−0.10	0.07	1.91	0.167	0.91	0.79	1.04	−67.61
	Experience	0.22	0.15	1.98	0.159	1.24	0.92	1.67	−66.62
Model Fit Statistics: AIC = 139.25, BIC = 148.78, Nagelkerke’s R^2^ = 0.04 || AUC Load = 0.44, AUC Experience = 0.57, Δ AUC = 0.14, *p* = **0.060**									
Upper limb	SMI	0.17	0.08	4.49	**0.034**	1.18	1.01	1.38	−79.62
	BMI	0.07	0.08	0.91	0.341	1.08	0.92	1.26	−79.16
Model Fit Statistics: AIC = 164.33, BIC = 173.86, Nagelkerke’s R^2^ = 0.08 || AUC SMI= 0.67, AUC BMI = 0.59, Δ AUC = −0.09, *p* = 0.115									
Lower limb	Age	−0.29	0.11	6.53	**0.011**	0.75	0.60	0.94	−115.23
	BMI	0.08	0.06	2.01	0.157	1.09	0.97	1.22	−114.27
Model Fit Statistics: AIC = 234.45, BIC = 243.98, Nagelkerke’s R^2^ = 0.07 || AUC Age = 0.39, AUC BMI = 0.44, Δ AUC = 0.06, *p* = 0.361									
				**Females**	**team**				
H-n-tr	Experience	−0.65	0.39	2.86	0.091	0.52	0.24	1.11	−17.43
	BMI	0.09	0.14	0.46	0.500	1.10	0.84	1.43	−17.21
Model Fit Statistics: AIC = 40.43, BIC = 40.61, Nagelkerke’s R^2^ = 0.14 || AUC Expierience = 0.73, AUC BMI = 0.51, Δ AUC = −0.22, *p* = 0.173									
Upper limb	MFR	0.90	0.38	5.70	**0.017**	2.46	1.17	5.15	−29.43
	BMI	0.52	0.19	7.14	**0.008**	1.67	1.15	2.44	−24.12
Model Fit Statistics: AIC = 54.23, BIC = 60.41, Nagelkerke’s R^2^ = 0.27 || AUC MFR = 0.56, AUC BMI = 0.61, Δ AUC = −0.05, *p* = 0.786									
Lower limb	Age	−0.44	0.22	4.08	**0.043**	0.64	0.42	0.99	−37.95
	FMI	0.53	0.20	6.89	**0.009**	1.70	1.14	2.52	−33.21
Model Fit Statistics: AIC = 72.42, BIC = 78.60, Nagelkerke’s R^2^ = 0.26|| AUC Age = 0.61, AUC FMI = 0.68, Δ AUC = −0.07, *p* = 0.540									

**Table 5:** Comparison of predictor effects (Beta and OR) across models in male and female athletes. Significant coefficients are presented in bold.

				Beta		OR	
Model 1	Variable 1	Model 2	Variable 2	Z-Score	*p*	Z-Score	*p*
**Males**							
Upper_Individuals	Age	Lower_Individuals	Age	−0.54	0.591	−0.58	0.561
Upper_Individuals	Age	Upper_Team	Age	−3.66	**0.000**	−3.76	**0.000**
Lower_Individuals	Age	Upper_Team	Age	−3.54	**0.000**	−3.61	**0.000**
**Females**							
Upper_Individuals	SMI	Lower_Individuals	Age	3.38	**0.001**	3.25	**0.001**
Upper_Individuals	SMI	Upper_Team	MFR	−1.88	0.060	−1.90	0.057
Upper_Individuals	SMI	Upper_Team	BMI	−1.70	0.090	−1.67	0.095
Upper_Individuals	SMI	Lower_Team	Age	2.61	**0.009**	2.63	**0.009**
Upper_Individuals	SMI	Lower_Team	FMI	−1.67	0.095	−1.68	0.093
Lower_Individuals	Age	Upper_Team	MFR	−3.01	**0.003**	−3.01	**0.003**
Lower_Individuals	Age	Upper_Team	BMI	−3.69	**0.000**	−3.58	**0.000**
Lower_Individuals	Age	Lower_Team	Age	0.61	0.542	0.64	0.521
Lower_Individuals	Age	Lower_Team	FMI	−3.59	**0.000**	−3.52	**0.000**
Upper_Team	MFR	Upper_Team	BMI	0.89	0.371	0.91	0.361
Upper_Team	MFR	Lower_Team	Age	3.05	**0.002**	3.08	**0.002**
Upper_Team	MFR	Lower_Team	FMI	0.86	0.389	0.86	0.389
Upper_Team	BMI	Lower_Team	Age	3.30	**0.001**	3.30	**0.001**
Upper_Team	BMI	Lower_Team	FMI	−0.04	0.971	−0.06	0.949
Lower_Team	Age	Lower_Team	FMI	−3.26	**0.001**	−3.28	**0.001**

## Data Availability

The data presented in this study are available upon request from the author.
